# Spontaneous pneumothorax caused by an inflammatory myofibroblastic tumor-like lesion in a 14-year-old girl: a case report

**DOI:** 10.1186/s40792-020-00873-2

**Published:** 2020-05-24

**Authors:** Hisayuki Miyagi, Daisuke Ishii, Masatoshi Hirasawa, Shunsuke Yasuda, Naohisa Toriumi, Takeo Sarashina, Mishie Tanino, Mio Tanaka, Yukichi Tanaka, Kazutoshi Miyamoto

**Affiliations:** 1 Division of Pediatric Surgery, Department of Surgery, Asahikawa Medical University, 2-1-1-1 Midorigaoka Higashi, Asahikawa, 078-8510 Japan; 2 Respiratory Center, Asahikawa Medical University, 2-1-1-1 Midorigaoka Higashi, Asahikawa, 078-8510 Japan; 3 Department of Pediatrics, Asahikawa Medical University, 2-1-1-1 Midorigaoka Higashi, Asahikawa, 078-8510 Japan; 4 Department of Pathology, Asahikawa Medical University, 2-1-1-1 Midorigaoka Higashi, Asahikawa, 078-8510 Japan; 5 Department of Pathology, Kanagawa Children’s Medical Center, Yokohama, 232-8555 Japan

**Keywords:** Spontaneous pneumothorax, Catamenial pneumothorax, Inflammatory myofibroblastic tumor (IMT), Anaplastic lymphoma kinase (ALK)

## Abstract

**Background:**

Spontaneous pneumothorax occurs more often in younger, slim, and shallow-chested men. Although less common, differential diagnoses for secondary pneumothorax in children are asthma, emphysematous blebs, catamenial pneumothorax, and others. We report a patient who presented with pneumothorax and was found to have an inflammatory myofibroblastic tumor (IMT)-like lesion, and present a review of the related literature.

**Case presentation:**

A 14-year-old girl visited her physician for chest pain that developed while exercising. Although chest drainage was performed, the symptoms associated with a collapsed lung did not improve, and she was referred to our hospital. Computed tomography revealed the presence of a 19 × 17-mm cyst with a thick wall in the apex of the right lung. She was tested for infectious diseases, namely tuberculosis, but the results were not definitive. Catamenial pneumothorax was also suspected because she was menstruating when she presented to our hospital. As a therapeutic diagnosis, we performed a thoracoscopic partial resection of the right upper lobe of the lung. Three small openings were identified inside the cyst, suggesting connection with the bronchiole. The lesion was pathologically diagnosed as an IMT-like lesion. Considering the progress so far, we considered that the final diagnosis to be an IMT. The patient was discharged on postoperative day 3, and we have followed her for the past 6 months with no local recurrence or metastasis.

**Conclusions:**

IMT is not uncommon in children. Therefore, this lesion should be considered as a possible diagnosis if children and young adults develop spontaneous pneumothorax.

## Background

Spontaneous pneumothorax occurs more often in the late teens, twenties, and thirties and more often in thin, shallow-chested men, and is usually classified as either traumatic or spontaneous. Trauma-related pneumothorax can be iatrogenic or accidental, and spontaneous pneumothorax can be primary or secondary. Although less common, differential diagnoses for secondary pneumothorax in children are asthma, emphysematous blebs, pulmonary tuberculosis, Langerhans cell histiocytosis, catamenial pneumothorax, Marfan syndrome, Ehlers–Danlos syndrome, Birt–Hogg–Dube syndrome, and angiosarcoma metastasis to the lung.

We report a patient who presented with pneumothorax and was found to have an inflammatory myofibroblastic tumor (IMT)-like lesion. We also reviewed the relevant literature.

## Case presentation

**Fig. 1:**
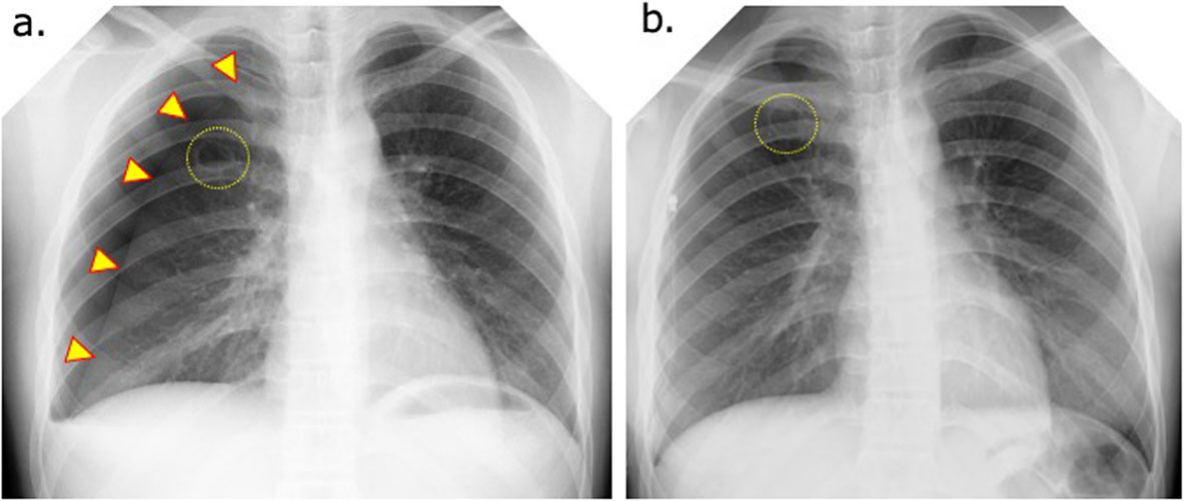
Chest X-ray at the patient’s first consultation. **a** Right pneumothorax is seen (yellow arrowheads). **b** At the previous hospital, placement of a thoracic drain for right pneumothorax did not improve the pneumothorax. Cyst wall thickening is visible in the upper right lung field (dashed yellow circle)

**Fig. 2:**
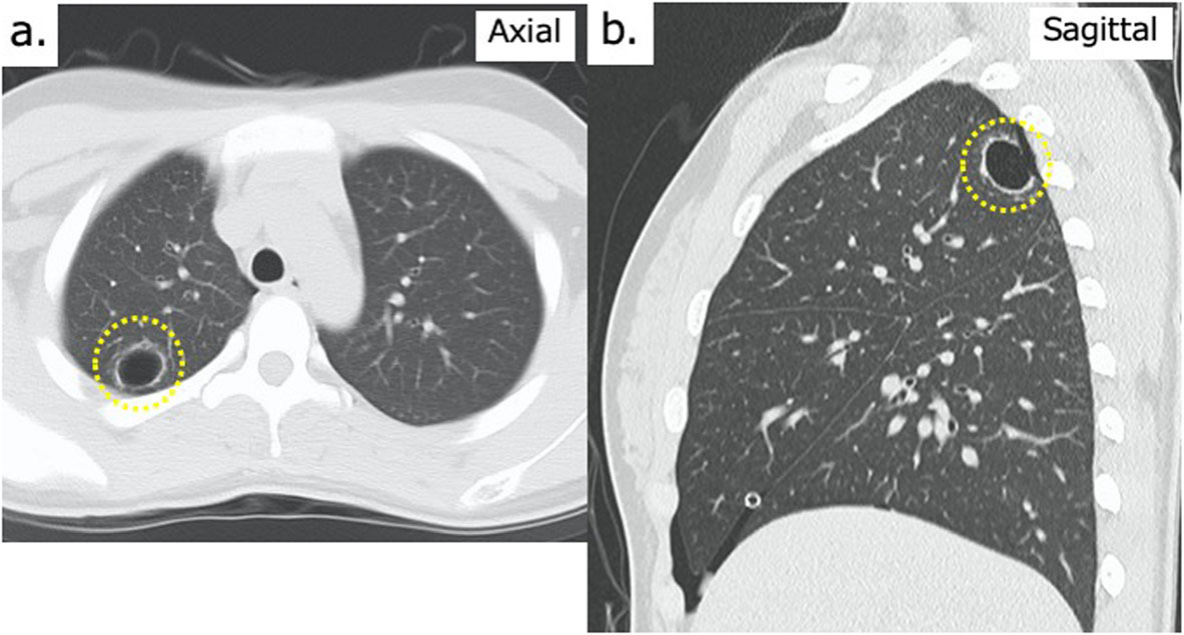
Preoperative chest computed tomography images. **a** Axial view showing a 19 × 17-cm cystic lesion in the right upper lobe. Thickening of the cyst wall is visible (dashed yellow circle). **b** Sagittal view showing a cyst located posterior to the apex of the right lung (S1-2)

The patient was a 14-year-old girl who visited her physician for chest pain that developed while exercising. Upon detailed examination, right-sided pneumothorax was identified, and chest drainage was performed (Fig. 1). However, the symptoms associated with a collapsed lung did not improve, and she was referred to our hospital. Computed tomography revealed the presence of a 19 × 17-mm cyst with a thick wall in the apex of the right lung (Fig. 2). She was tested for infectious diseases, namely tuberculosis, but the results were not definitive (Table 1). Catamenial pneumothorax was also suspected because she was menstruating when she presented to our hospital. As a therapeutic diagnosis, we performed a thoracoscopic partial resection of the right upper lobe of the lung.

**Table 1:** The patient’s laboratory data at admission

**TP**	7.7	g/dl	**WBC**	6890	/μl
**Alb**	4.8	g/dl	**Hb**	13.2	g/dl
**T-Bil**	0.5	mg/dl	**Ht**	39.0	%
**AST**	20	U/l	**PLT**	40.3 × 10^4^	/μl
**γGT**	29	U/l			
**AMY**	65	U/l			
**BUN**	9.6	mg/dl			
**Cre**	0.43	mg/dl			
**UA**	3.6	mg/dl			
**Na**	138	mEq/l			
**K**	4.1	mEq/l			
**Cl**	103	mEq/l			
**Ca**	9.7	mg/dl			
**CRP**	< 0.10	mg/dl			
**LDH**	144	U/l			
**CK**	54	U/l			
**T-SPOT tuberculosis-specific IFNγ**			(−)		

### Operation

**Fig. 3:**
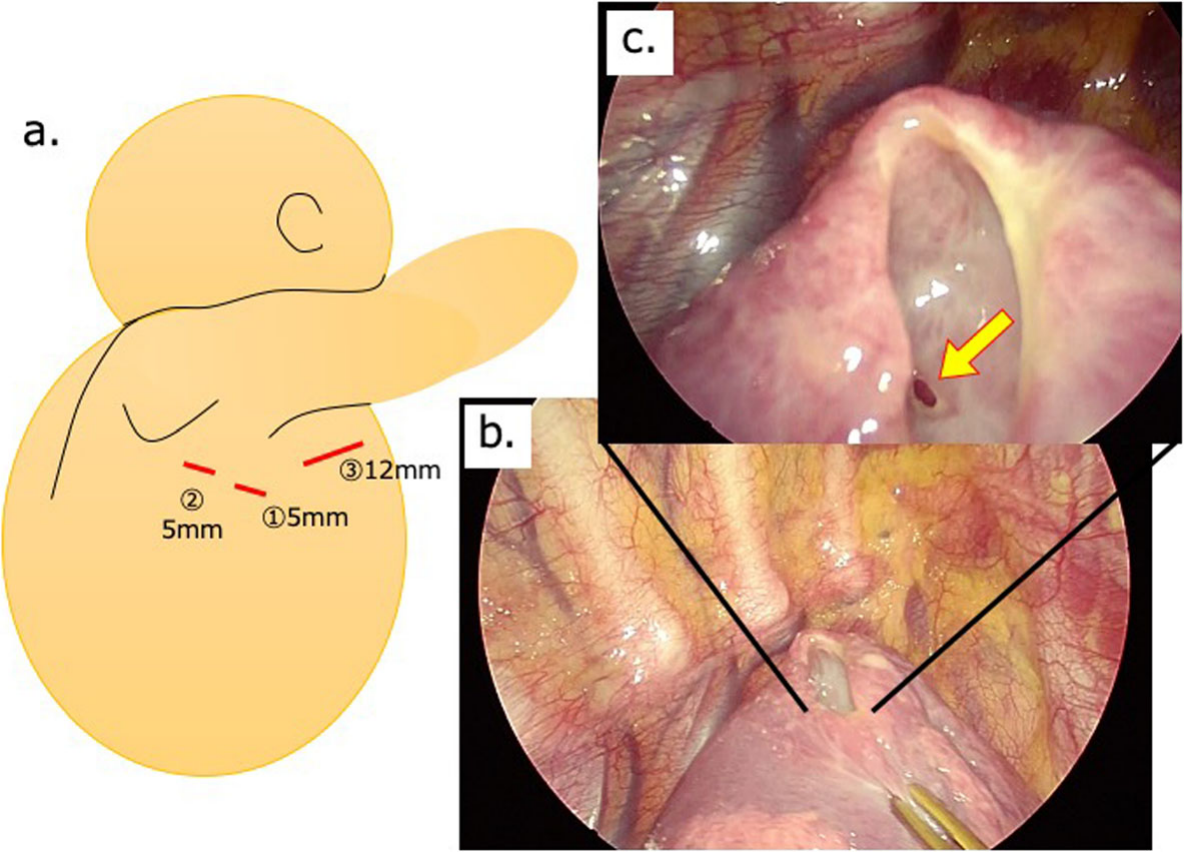
Operative findings. **a** Thoracoscopic partial resection of the right upper lobe showing the three ports: (1), (2), and (3). We inserted a 5-mm port on the mid-axillary line at the sixth intercostal space (1), followed by a 5-mm port inserted on the posterior axillary line at the sixth intercostal space (2), and a 12-mm port on the anterior axillary line at the fourth intercostal space (3). **b** The cyst wall is partially ruptured in the upper right lobe (S1-2). **c** An opening to the bronchiole was confirmed in the cyst wall (yellow arrow)

With the patient being under general anesthesia, at the left lateral position, we inserted a 5-mm port on the mid-axillary line at the sixth intercostal space, followed by a 5-mm port inserted on the posterior axillary line at the sixth intercostal space, and a 12-mm port on the anterior axillary line at the fourth intercostal space. The thoracic cavity was thoroughly observed via thoracoscopy. No blueberry spots were found on the visceral pleura or the diaphragm (catamenial pneumothorax was considered negative). A ruptured cyst with a thick wall was found at the apex of the right lung, and three small openings were identified inside the cyst, suggesting communication with the bronchiole. The margin was sufficiently secured from the cyst, the lesion was excised using an Endo GIA^TM^ ultra-universal stapler (purple) (Covidien Surgical, Norwalk, CT), and the specimen was collected with an Endo Catch^TM^ Gold device (Covidien Surgical). A 20-Fr. thoracic drain was placed at the apex of the right lung through the first port to complete the operation. The operation time was 83 min, and the blood loss volume was almost negligible (Fig. 3).

### Pathological diagnosis

**Fig. 4:**
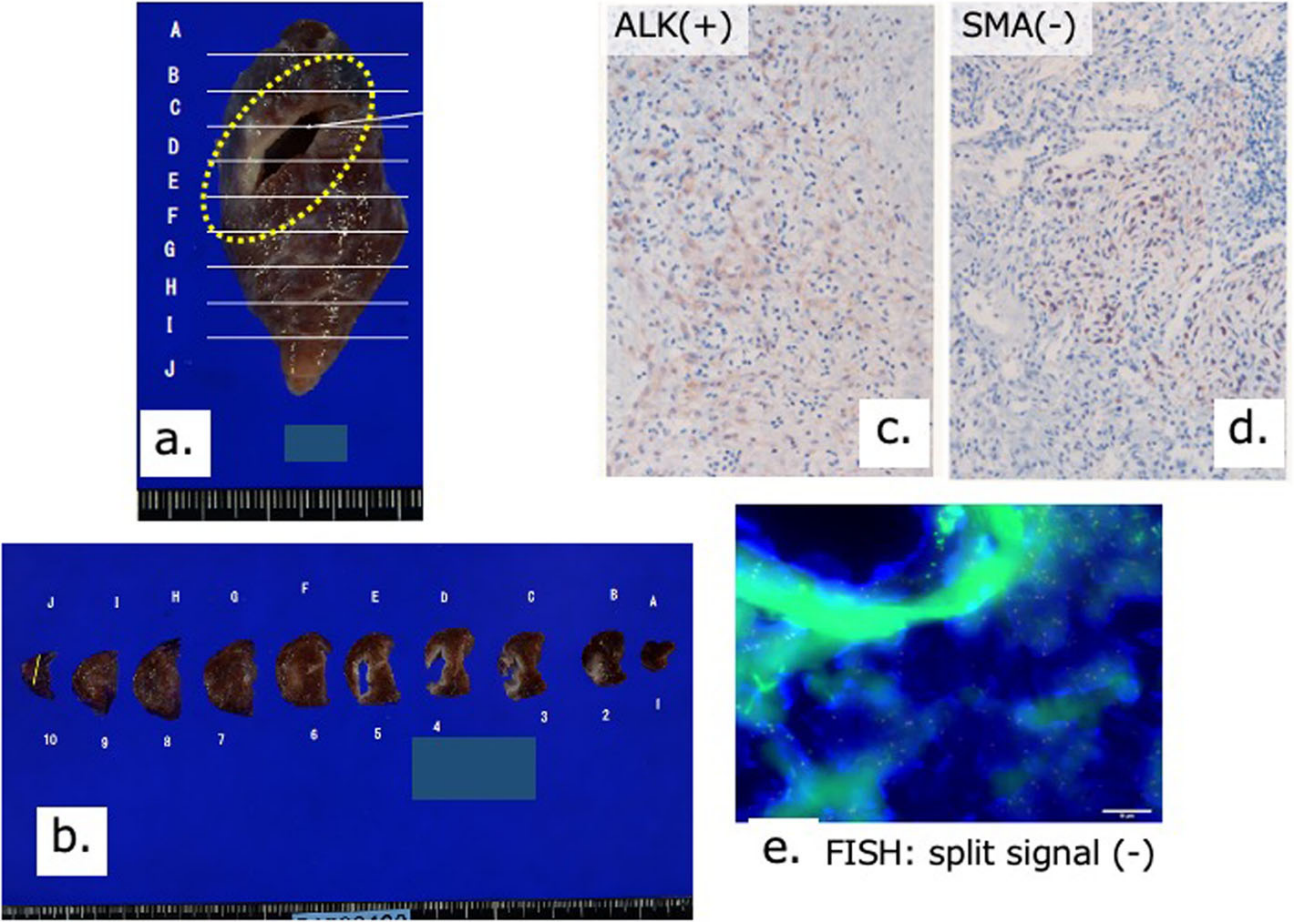
Pathological findings. **a** Macroscopic pathological findings. The area inside the dashed yellow circle indicates the ruptured cyst. **b** Macroscopic pathological findings. The cyst wall is thick and ruptured dorsally. **c**–**e** Immunohistochemical findings and results of FISH testing. Because SMA staining was negative and ALK reconstitution could not be confirmed by FISH, it was difficult to confirm the lesion as IMT. The final diagnosis in our patient was an IMT-like lesion. FISH, fluorescence in situ hybridization; SMA, smooth muscle actin; IMT, inflammatory myofibroblastic tumor; ALK, anaplastic lymphoma kinase

Pathological findings around the cyst wall included the infiltration of inflammatory cells, proliferation of fibroblasts, and the slightly dense distribution of mildly atypical spindle cells. Tumor cells proliferating around the cyst wall were anaplastic lymphoma kinase (ALK) (+), pan-tropomyosin receptor kinase (−), smooth muscle actin (SMA) (−), transducer-like enhancer of split 1 (−), BCL6 corepressor gene (−), and alpha-internexin (−), using immunohistochemistry. In addition, no ALK split signal was observed using fluorescence in situ hybridization (Fig. 4c–e). Differential diagnoses were IMT and congenital peribronchial myofibroblastic tumor. However, congenital peribronchial myofibroblastic tumor is often identified and operated during infancy and has a different histopathology from IMT. Because SMA was negative in this case and ALK rearrangement could not be determined by fluorescence in situ hybridization, it was difficult to confirm IMT; nevertheless, the pathological diagnosis was an IMT-like lesion in our patient. Not all diagnostic materials were available for pathological diagnosis, so we based the final diagnosis of IMT on the clinical course including the patient background. The patient was discharged on postoperative day 3. We have followed her for the past 6 months and have not observed local recurrence or metastasis.

## Discussion

The differential diagnoses for pneumothorax in children are asthma, emphysematous blebs, pulmonary tuberculosis, Langerhans cell histiocytosis, catamenial pneumothorax, Marfan syndrome, Ehlers–Danlos syndrome, Birt–Hogg–Dube syndrome, angiosarcoma metastasis to the lung, and others. To the best of our knowledge, no reports have discussed patients with IMT presenting with spontaneous pneumothorax.

An IMT is a mesenchymal neoplasm of intermediate malignancy (rarely metastasizing) according to the 2013 World Health Organization classification [1], which may develop in almost every organ [2]. Some authors reported that an IMT in the lung is rare, with an incidence of 0.04% [3] to 0.3% [4]. Sex, race, and geographical location appear to play no role in the occurrence of IMT [4]. Cerfolio et al. reported that the median age of their patients was 47 years (range, 5–77 years) [4]. Others reported that IMT occurs in various anatomical locations, among which the lung is the most common site followed by the abdomen and retroperitoneum. IMT is generally more common in children and young adults and accounts for < 1% of all lung tumors [5]. Although IMT is defined as an intermediate tumor with the potential for local recurrence or metastasis, distant metastasis, most likely as extrapulmonary tumors, is seldom seen [6].

Tanaka et al. emphasized that IMTs are one of the most common primary lung tumors in children [7]. Although IMTs account for 20–50% of all pediatric primary lung tumors, they represent less than 1% of lung tumors in adults [8, 9]. Because of uncertainty regarding the true biological origin of these tumors, other terms have been used to refer to IMTs such as plasma cell granuloma, xanthogranuloma, inflammatory myofibroblastic tumor, inflammatory pseudotumor, fibroxanthoma, and fibrous histiocytoma [8, 10]. We emphasize that IMT of the lung is not uncommon in children.

### Clinical features

The chief complaint in patients with pulmonary IMT is usually nonspecific, which makes the diagnosis difficult to confirm. IMT is asymptomatic in many patients, whereas respiratory tract infection-related symptoms, such as cough, shortness of breath, chest pain, or complaints of fatigue, fever, and weight loss, are common in others. There are no reports of IMT in children with pneumothorax. It is difficult to consider IMT in the lung for preoperative diagnosis as in our case, and imaging findings might be one of the diagnostic cofactors that consider IMT for differential diagnosis. Although there are no characteristic imaging findings in IMT, calcification is relatively common in IMT occurring in children. Hamartoma is often suspected if calcification is seen in a mass lesion of the lung, but this is rare in children. Therefore, IMT is one of the differential diseases when a mass lesion suspected of calcification is found in children [11].

Laboratory examinations in most instances of IMT are normal, but anemia, thrombocytopenia, and an elevated sedimentation rate may be revealed. Radiographically, pulmonary IMT usually occurs as a single nodule or a mass with clear boundaries and slight to moderate homogeneous contrast enhancement; however, occasionally, IMT may mimic malignant tumors [5]. According to previous reports, the incidence of multiple nodules accounts for 5% of pulmonary IMTs [12]. In the current case, the patient complained of dyspnea, and imaging findings showed a cystic lesion with thickened walls on chest X-ray and chest computed tomography.

### Pathological findings

IMTs are rare neoplasms composed histopathologically of differentiated myofibroblastic spindle cells accompanied by inflammatory cells [13]. Rearrangements involving the ALK locus on chromosome 2p23 have been discovered in 50% of patients with IMT [14, 15].

Pulmonary IMT rarely occurs in the neonatal or infantile period [16, 17], although IMT is one of the most common pulmonary tumors in older children [7]. Histological examination of typical pediatric pulmonary IMTs reveals fascicles of myofibroblastic cells admixed with an inflammatory infiltrate consisting of lymphocytes, plasma cells, and eosinophils [7]. Histological analysis of these tumors in older children reveals fascicles of spindle cells, which are consistent with IMT, but with little inflammatory infiltration [18, 19]. Coffin et al. reported the immunohistochemical analysis of IMT in which immunoreactivity for muscle-specific actin (89%) and smooth muscle actin (92%) varied from a focal to a diffuse pattern in the cytoplasm of spindle cells; generally, smooth muscle actin staining was more prominent than muscle-specific actin reactivity [6].

### Treatment

Liu et al. [5] reported that, in general, IMT patients who are candidates for surgical resection have a favorable prognosis, with 5- and 10-year survival rates of 91% and 77%, respectively [20]. Nevertheless, approximately 8% of pulmonary IMTs without indications for surgery have a continuous growth pattern, with 5% of patients at risk of developing distant metastasis [18]. Distant metastases appear mainly in ALK-negative IMTs, but local recurrence occurs regardless of ALK expression [6]. In a study involving 44 patients, Kovach et al. demonstrated an 8% recurrence rate with a primary surgical approach and proposed surgical resection of all lesions if there are no contraindications related to the patient’s anatomy or morbidity [21].

Regarding inoperable patients, including patients with unresectable recurrence or metastasis, therapeutic approaches, such as steroids, nonsteroidal anti-inflammatory drugs, chemotherapy, and radiotherapy, provide unsatisfactory effects [5]. One report described using steroids or nonsteroidal anti-inflammatory drugs with a combination of epirubicin, dacarbazine, and docetaxel or vinorelbine plus methotrexate, programmed cell death ligand-1, and apatinib [5].

## Conclusion

In conclusion, IMTs are not uncommon in children; therefore, IMT should be considered as a diagnosis if children or young adults develop spontaneous pneumothorax.

## Data Availability

Not applicable.

## Abbreviations

ALK: Anaplastic lymphoma kinase
IMT: Inflammatory myofibroblastic tumor
SMA: Smooth muscle actin
